# Increased plasma level of terminal complement complex in AMD patients: potential functional consequences for RPE cells

**DOI:** 10.3389/fimmu.2023.1200725

**Published:** 2023-06-08

**Authors:** Catharina Busch, Saskia Rau, Andjela Sekulic, Luce Perie, Christian Huber, Miranda Gehrke, Antonia M. Joussen, Peter F. Zipfel, Gerhild Wildner, Christine Skerka, Olaf Strauß

**Affiliations:** ^1^ Department of Ophthalmology, University Hospital Leipzig, Leipzig, Germany; ^2^ Experimental Ophthalmology, Department of Ophthalmology, Charité - Universitätsmedizin Berlin, Corporate Member of Freie Universität, Berlin Institute of Health, Humboldt-University, Berlin, Germany; ^3^ Department of Infection Biology, Leibniz Institute for Natural Product Research and Infection Biology, Hans-Knoell-Institute, Jena, Germany; ^4^ Section of Immunobiology, Department of Ophthalmology, University Hospital, Ludwig-Maximilians-Universität (LMU) Munich, Munich, Germany; ^5^ Institute of Microbiology, Friedrich-Schiller-University, Jena, Germany

**Keywords:** retinal pigment epithelium, terminal complement complex, age-related macular degeneration, AMD serum, genetic risk factors

## Abstract

**Purpose:**

Polymorphisms in complement genes are risk-associated for age-related macular degeneration (AMD). Functional analysis revealed a common deficiency to control the alternative complement pathway by risk-associated gene polymorphisms. Thus, we investigated the levels of terminal complement complex (TCC) in the plasma of wet AMD patients with defined genotypes and the impact of the complement activation of their plasma on second-messenger signaling, gene expression, and cytokine/chemokine secretion in retinal pigment epithelium (RPE) cells.

**Design:**

Collection of plasma from patients with wet AMD (n = 87: 62% female and 38% male; median age 77 years) and controls (n = 86: 39% female and 61% male; median age 58 years), grouped for risk factor smoking and genetic risk alleles *CFH* 402HH and *ARMS2* rs3750846, determination of TCC levels in the plasma, *in vitro* analysis on RPE function during exposure to patients’ or control plasma as a complement source.

**Methods:**

Genotyping, measurement of TCC concentrations, ARPE-19 cell culture, Ca^2+^ imaging, gene expression by qPCR, secretion by multiplex bead analysis of cell culture supernatants.

**Main outcome measures:**

TCC concentration in plasma, intracellular free Ca^2+^, relative mRNA levels, cytokine secretion.

**Results:**

TCC levels in the plasma of AMD patients were five times higher than in non-AMD controls but did not differ in plasma from carriers of the two risk alleles. Complement-evoked Ca^2+^ elevations in RPE cells differed between patients and controls with a significant correlation between TCC levels and peak amplitudes. Comparing the Ca^2+^ signals, only between the plasma of smokers and non-smokers, as well as heterozygous (*CFH 402YH*) and *CFH 402HH* patients, revealed differences in the late phase. Pre-stimulation with complement patients’ plasma led to sensitization for complement reactions by RPE cells. Gene expression for surface molecules protective against TCC and pro-inflammatory cytokines increased after exposure to patients’ plasma. Patients’ plasma stimulated the secretion of pro-inflammatory cytokines in the RPE.

**Conclusion:**

TCC levels were higher in AMD patients but did not depend on genetic risk factors. The Ca^2+^ responses to patients’ plasma as second-messenger represent a shift of RPE cells to a pro-inflammatory phenotype and protection against TCC. We conclude a substantial role of high TCC plasma levels in AMD pathology.

## 1 Introduction

Age-related macular degeneration (AMD) is one of the major causes of vision loss in industrialized countries (1). The disease shows two clinically defined end stages. One is the loss of cells of the retinal pigment epithelium (RPE) and photoreceptors, which progresses slowly. This form, the so-called “dry AMD” or geographic atrophy, accounts for approximately 80% of the cases but due to its low progression for approximately 20% of blindness. The other end stage is characterized by the proliferation of endothelial cells, resulting in pathologic neo-angiogenesis that leads to bleeding due to low grade of blood vessel differentiation and thus too fast loss of vision (2). This end stage, the so-called choroidal neovascularization or “wet AMD”, represents the majority of cases of blindness. AMD is a multifactorial disease for which environmental risk factors like smoking and genetic risk factors like polymorphisms in genes such as *ARMS2* (age-related macular susceptibility-2) or genes of the complement components are known (3–5).

The identification of complement deposits in drusen as well as polymorphisms in complement genes as risk factors suggested that the complement system is associated with the development of AMD. Recent reports confirmed that an over-activated complement system due to reduced regulation represents a risk for AMD (3, 4, 6–10). However, recent clinical trials failed to establish a complement inhibition-based therapy for the dry form of AMD (11–13). Therefore, the role of activated complement proteins is more complex, and the chronic AMD-associated complement activity is still not fully understood (12).

Another reason why complement-targeting clinical trials failed might be the fact that the systemic over-activity of the complement cascade has a life-long impact on the retina. This would explain why acute intervention into complement activity might not immediately reverse chronic events that have affected the outer retina for decades (12). Complement activation may have a lifelong systemic impact on the outer retina with strong local pathologic changes. The impact of the end product of the complement cascade, the terminal complement complex (TCC; also termed C5b-9), is not clear so far. The TCC is a complex of five different proteins (C5b, C6, C7, C8, and C9). When this complex is formed on a cell surface, several C9 components are aggregated and form a ring, inserting a pore into the cell membrane, which leads to cell lysis and is also called membrane attack complex (MAC). Indeed, depending on age, AMD status, and risk allele, TCC accumulates in the outer retina. TCC is increasingly found in the tissue complex of the choroid, Bruch’s membrane, and RPE during its lifetime (14–17). AMD patients with C9 risk alleles show increased plasma levels of C9 or TCC compared to healthy non-carriers (18–20), although no differences were reported among AMD with or without C9 risk alleles. This might be explained by the fact that AMD patients with the absence of C9 risk alleles generally display increased systemic complement activities (21–23). In the plasma of patients carrying various C9 risk alleles, higher levels of C9 but no differences in TCC plasma levels were found (19). When comparing different C9 risk alleles, it appeared that some are associated with higher C9 or TCC plasma levels (20). This suggests an important role of TCC in AMD pathology.

Acting as MAC, the consequence of TCC accumulation in the outer retina would destabilize RPE cells, which results in the loss of RPE cells (24–26). The RPE itself is a close interaction partner of the light-sensitive photoreceptors, supporting their function and even contributing to visual function (27). Thus, the loss of RPE cells in AMD ultimately causes loss of photoreceptors and blindness. Several observations about the general nature of TCC effects and the concept of the specific complement impact on the RPE are still unclear. In the RPE, the human plasma-derived complement ignites a well-orchestrated Ca^2+^ signal that depends on the activation of endogenously expressed Ca^2+^ and K^+^ channels. The induction of an unspecific pore could not be detected in measurements of the membrane conductance by means of the patch-clamp technique (28). The different complement activation products’ consequence is that the RPE cells change their gene expression pattern toward a more pro-inflammatory profile in response to anaphylatoxins via the Akt–kinase pathway and the transcription factor FoxP3 (29–33).

TCC’s influence on gene expression emerged as the concept of sub-lytic MAC in studies that investigated the effects of different TCC concentrations and combinations at various cell types (25, 34–39). The concept bases on the ability of cells to remove MAC from the plasma membrane. As long as the cell’s ability to remove MAC from the plasma membrane can cope with the rate of extracellular TCC formation and membrane insertion, the cell will not undergo lysis (35). Soluble TCC is generally accepted as a marker of *in vivo* complement activation (40). These studies defined TCC concentrations that do not lyse cells but lead to functional changes as sub-lytic (34, 35, 37, 38). The functional changes require intracellular signaling such as an increase in intracellular Ca^2+^, activation of different types of kinases, and even the activation of G proteins (25, 34, 35, 37–39, 41). The activation of these intracellular signaling pathways then lead to secondary effects such as changes in the gene expression profile and represent the basic sub-lytic MAC effects onto cellular functions. Several studies demonstrated functional changes of the RPE by sub-lytic MAC, mainly in the secretory activity of, e.g., VEGF-A or MCP-1, or reduced stress response (5, 30, 33, 42, 43). This might be explained by CD59, which inactivates MAC formation (7, 44) and represents a target for further therapeutic approaches (26). However, Cipriani et al. found no risk association of TCC regulators CD46, CD55, and CD59 with AMD, especially not for CD59 (45).

As a consequence, the enhanced generation of TCC in AMD patients might not directly lead to RPE cell death but promote a change of the RPE’s immunogenic phenotype toward low-grade chronic inflammation. To test this concept, we have used human plasma from AMD patients with defined genetic risk factors as a complement source to test RPE cell reactions to dysregulated complement. Depending on the patients’ genotype, these plasma contained different levels of TCC and caused different Ca^2+^ signals as well as different regulatory effects on gene expression. Our hypothesis is that TCC complexes in AMD patients generate Ca^2+^ signals with profound physiological changes in the RPE.

## 2 Materials and methods

### 2.1 Blood samples and determination of TCC concentration

A total of 87 AMD patients with wet AMD (62% female and 38% male) with a mean age of 77.9 years (median = 77 years) and 86 age-matched controls (39% female and 61% male) with a mean age of 55.1 years (median = 58) were included; further information is presented in the tables of **Supplementary Material Suppl. 1**, **2**. The samples were anonymized. Blood samples were collected with EDTA and centrifuged, and plasma was stored at −80°C. The concentration of TCC was determined by ELISA as previously described (46). In brief, plasma samples were pre-incubated (37°C; 15 min) and incubated on lipopolysaccharide (LPS)-coated plates. Plates were analyzed at 450-nm wavelength using TCC mAB from TECOmedical (Sissach, Switzerland). The polymorphisms in this patient cohort were determined as described earlier for *ARMS* (47) and *CFH* (9, 48).

### 2.2 RPE cell culture

For our study, the ARPE-19 cell line was used, which shows limitations for conclusions about native RPE cells. Here, these cells were used to monitor the biological activity of activated complement proteins in human plasma rather than to investigate specific RPE cell functions. The cells were grown on glass coverslips to confluence prior to the Ca^2+^ imaging experiments. The culture conditions were using Dulbecco’s modified Eagle medium (DMEM)/F12 (Thermo Fisher, Darmstadt, Germany) with GlutaMAX (stable glutamine) supplemented with 10% fetal calf serum (FCS) and 50 U penicillin/50 mg streptomycin at 37°C and 5% CO_2_. The medium was changed twice a week.

### 2.3 Ca^2+^ imaging experiments

As a readout of biological responses to the activated complement, intracellular free Ca^2+^ as a second messenger was measured by means of Ca^2+^ imaging techniques based on fluorescence microscopy using the Ca^2+^-sensitive fluorescence dye fura-2. Confluent ARPE-19 cells grown on glass coverslips were incubated in the membrane-diffusible fura-2 ester fura-2-AM (2 µM) for 40 min at room temperature. Fura-2 loaded cells were mounted onto the stage of a Zeiss Axiovert 40 CFL inverted microscope (Carl Zeiss, Oberkochen, Germany) with an attached Visitron Polychromator (Visitron Systems, Puchheim, Germany) and a high-sensitivity color event camera (CED) camera (CoolSNAP EZ, Photometrics, Tucson, AZ, USA). Experiments were conducted using the MetaFlour software (Visitron Systems, Puchheim, Germany). The cells were bathed in an extracellular solution containing (mM): 138 NaCl, 5.8 KCl, 0.41 MgSO_4_, 0.48 MgCl_2_, 0.95 CaCl_2_, 4.17 NaHCO_3_, 1.1 NaH_2_PO_4_, and 25 HEPES; pH = 7.2 adjusted with Tris base. Fura-2 fluorescence was measured at 505-nm wavelength and excited by the wavelengths 340 and 380 nm through a semiconducting mirror. The changes in intracellular free Ca^2+^ were given as fluorescence ratio (dF/F) from baseline (ddF/F) between the excitation wavelengths 340 and 380 nm. Human plasma samples were directly applied to the cells on the stage of the microscope at a concentration of 20%. C6-depleted plasma was purchased from CompTech (Complement Technology, Tyler, TX, USA).

### 2.4 qPCR

In order to assess the functional consequences of complement-activated intracellular Ca^2+^ signaling, gene expression activity was measured by quantification of mRNA in ARPE-19 cells. Confluent ARPE-19 cells were exposed to different human plasma and in some conditions in combination with L-type channel blocker nifedipine (10 µM). After cell harvest, RNA isolation and cDNA synthesis were performed using RNeasy Mini and QuantiTect Reverse Transcription Kit (Qiagen, Hilden, Germany). mRNA levels were measured in triplicates for the target genes and GAPDH as a housekeeping gene for standardization in a Rotor-Gene Q (Qiagen, Hilden, Germany) by using the Rotor-Gene SYBR Green PCR Kit (Qiagen, Hilden, Germany); primer sequences are shown in **Table 1**. The mRNA levels of the target genes were calculated and presented as a comparative CT (threshold cycle, ΔΔCT) method using Rotor-Gene Q software 2.2.3 (Qiagen) (49). Of note, the primers for *CFH*, IL-1β, and CD46 showed reduced efficacy in the presence of dimethyl sulfoxide (DMSO) needed for the solubilization of nifedipine; thus, respective results from nifedipine-treated cultures could not be obtained.

**Table 1:** Primer sequences.

Gene	Forward sequence	Reverse sequence
C3	TTCCGATTGAGGATGGCTCG	ATGTCACTGCCTGAGTGCAA
C3aR	GGCTGTCTTTCTTGTCTGCTG	GACTGCCTTGCTTTCTTCCTAA
C5	ACACTGGTACGGCACGTATG	GGCATTGATTGTGTCCTGGG
C5aR	ThermoFisher TaqMan Primer Hs00704891_s1	
GAPDH	TCAACGACCACTTTGTCAAGCTCA	GCTGGTGGTCCAGGGGTCTTACT

### 2.5 Cytokine secretion of ARPE-19 cells in response to human plasma

ARPE-19 cells were grown in 96-well plates to confluency. Three days later, cultures were switched to serum-free DMEM/Ham’s F12 medium for 24 h before triplicate cultures were set up with 10% human plasma from smokers or non-smokers who were either homozygous (*CFH 402HH*) or heterozygous (*CFH 402YH*) or low-risk allele carriers (*CFH 402YY*), respectively, and incubated for another 3 days. Identical cultures were set up with 50 µM PI3 K-inhibitor LY294002 (Cayman Chemical, Ann Arbor, MI, USA). Plasma from age-matched, non-AMD donors without *CFH* mutations was used as the control.

Supernatants were collected after 24, 48, and 72 h; immediately shock frozen at −80°C; pooled in equal volumes before being tested for cytokines by human Bio-Plex bead analysis (Bio-Rad Laboratories, Inc., Hercules, CA, USA); and measured with a Bio-Plex 200 reader (Bio-Rad Laboratories, Inc.) according to the manufacturer’s instruction. Tested analytes were IL-1β, IL-1RA, IL-6, IL-8/CXCL8, IL-10, IL-12(p70), IFN-gamma, MCP-1/CCL2, and VEGF. Only results from those cytokines and chemokines that were secreted by the ARPE-19 cells are shown. Experiments were performed twice with ARPE-19 cells from different sources in different passages (as indicated, p6, p15, and p25) with comparable results. Data are shown as means + SE from triplicate cultures of one representative experiment with two different cell lines; experiments were performed twice.

### 2.6 Data analysis and statistical testing

All data are presented as mean values ± SEM or ± SD. Statistical significance was calculated using Mann–Whitney *U* test for Ca^2+^ imaging data and qPCR data (*p < 0.05, **p < 0.01, and ***p < 0.005). All calculations were performed in SPSS 26 and Excel 2010.

## 3 Results

**Figure 1:**
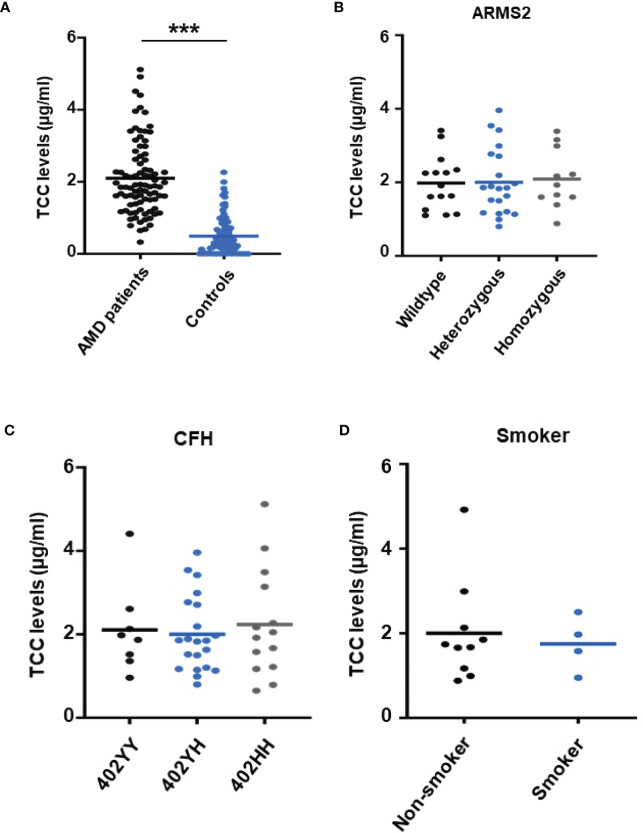
Plasma levels of terminal complement complex (TCC) in plasma from healthy donors and age-related macular degeneration (AMD) patients with AMD risk alleles. **(A)** Plasma levels of TCC (µg/ml) in AMD patients (n = 87) *vs.* healthy controls (n = 86) showing significantly higher TCC levels in AMD patients (*p* < 0.001). **(B)** TCC levels stratified for *Age-related maculopathy-susceptibility 2* (*ARMS2*) risk alleles, with all patients carrying one *Complement factor H* (*CFH 402YH*) risk allele. No significant difference in TCC levels among patients with no (wild type (WT), n = 15), one (heterozygous, n = 21), and two (homozygous, n = 11) *ARMS2* risk alleles existed. **(C)** TCC levels stratified for *CFH* risk alleles, with all patients carrying one *ARMS2* risk allele. No significant difference in TCC levels among patients with no (*CFH 402YY*, n = 8), one (*CFH 402YH*, n = 21), and two (*CFH 402HH*, n = 14) *CFH* risk alleles. The horizontal lines represent the means of the TCC levels. ***p < 0.001 (Mann–Whitney *U* test). **(D)** TCC levels stratified for the risk factor smoking and non-smoking among patients who do not carry one of the investigated genetic risk alleles. As the “heterozygous patients” are heterozygous for *CFH* (*CFH 402YH*) and *ARMS2*, the same set of plasma samples from these patients was used for the comparison with homozygous deficient *CFH* **(B)** and *ARMS2* **(C)** as well as with respective WT plasma.

To re-test the hypothesis of systemic complement activation in AMD, the TCC plasma levels in probes from AMD patients (the details of the patient cohort and controls in **Supplementary Material Tables S1, S2**) were compared with probes from age-matched controls (**Figure 1**) and found significantly, approximately five times, higher levels of TCC in AMD patients (0.44 ± 0.06 versus 2.15 ± 0.08 µg/ml; *p* < 0.001) (**Figure 1A**). The levels of TCC in healthy donors were in accordance with those that we published earlier (46). However, when comparing TCC concentrations in plasma from AMD patients carrying risk alleles, a different picture evolved. Comparing carriers of the low-risk allele of *ARMS2* versus high-risk allele carriers (*ARMS2* rs3750846) revealed no differences between low-risk and heterozygous or homozygous carriers (**Figure 1B**). Similar results were obtained among *CFH* polymorphism carriers with no differences between low-risk (*CFH 402YY*), heterozygous high-risk (*CFH 402YH*), or homozygous high-risk (*CFH 402HH*) allele carriers in AMD patients. Furthermore, no differences between the *ARMS2* and the *CFH* risk carriers were identified. The same applies to the environmental risk factor that we have analyzed in more detail, the smoking status (**Figure 1D**). In AMD patients without these genetic risk factors, no differences in the TCC concentrations were observed depending on their smoking status.

**Figure 2:**
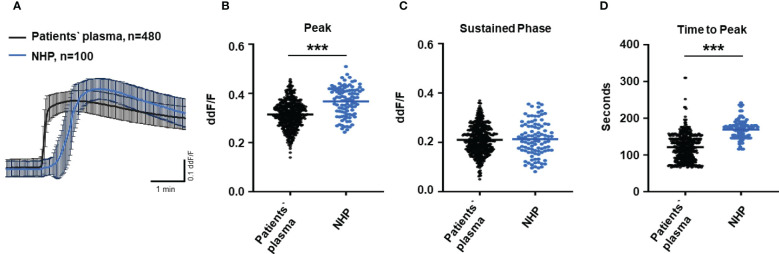
Changes in Ca^2+^ transients activated by C6-depleted plasma and normal human plasma (NHP) in ARPE-19 cells. **(A)** Ca^2+^ transients are given as differences to the baseline in fluorescence ratio between the two excitation wavelengths 340 and 380 nm; plasma concentrations were 10%. Data are mean ± SEM. **(B–D)** Ca^2+^ transient changes at the initial peak phase and the late sustained phase. C6-depleted plasma induced a significantly lower change in intracellular free Ca^2+^ compared to NHP. The horizontal line represents the mean change in Ca^2+^ transients from baseline. ***p < 0.001 (Mann–Whitney *U* test).

To test the biological relevance of increased TCC levels in the plasma of AMD patients, we used Ca^2+^ imaging techniques to explore the acute reaction of ARPE-19 cells on the patients’ plasma. Earlier studies showed that an increase in intracellular free Ca^2+^ that was evoked by exposure to human plasma as the source of complement reflects the concerted activity of all activated complement factors (28). Thus, changes were expected in intracellular Ca^2+^ transients by human plasma from AMD patients. As a first step, we investigated whether the Ca^2+^ response to human plasma was dependent on TCC in normal human plasma (NHP). We compared Ca^2+^ transients induced by C6-depleted plasma with those induced with NHP (**Figure 2**). Indeed, without C6, the plasma induced a Ca^2+^ response half in amplitude compared to that of the control plasma (**Figures 2A, B**). As the peak in C6-depleted plasma is much smaller, the peak in C6-depleted plasma is reached in a shorter time (**Figure 2C**). Furthermore, C6-depleted plasma failed to develop a sustained phase of an intracellular Ca^2+^ increase (**Figure 2D**).

**Figure 3:**
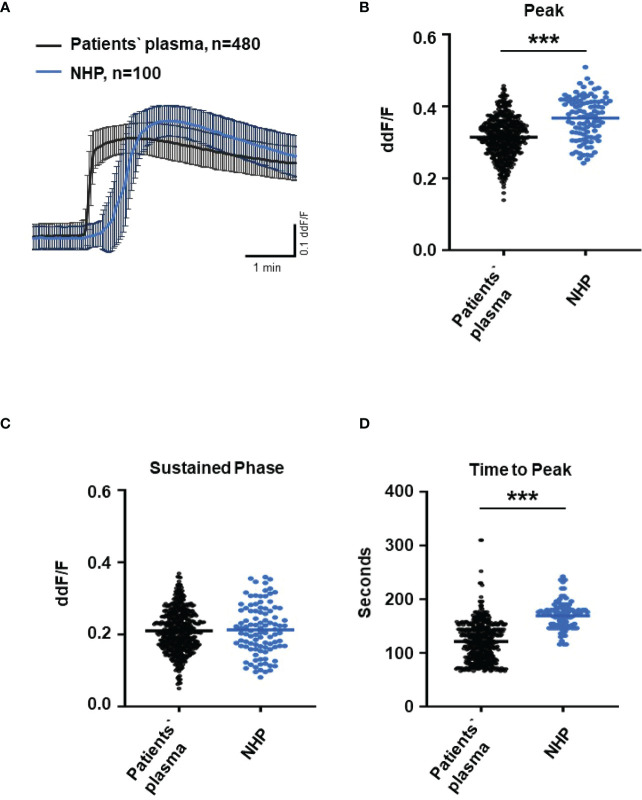
Changes in Ca^2+^ transients activated by plasma from age-related macular degeneration patients. **(A)** Ca^2+^ transients are given as differences to the baseline in fluorescence ratio between the two excitation wavelengths 340 and 380 nm. Experiments were conducted with plasma (10%) from 16 different patients (30 cells per patient) and normal human plasma (NHP) in ARPE-19 cells. Data are mean ± SD. **(B)** Ca^2+^ transient changes at the initial peak phase and the late sustained phase. NHP induced a significantly higher change in intracellular free Ca^2+^ in the initial phase compared to patients’ plasma. **(C, D)** Mean time until maximum Ca^2+^ transient change. Patients’ plasma induced significantly faster maximum Ca^2+^ transients to change compared to NHP. The horizontal line represents the mean change in Ca^2+^ transients from baseline **(B)** and mean time until peak **(C)**. ***p < 0.001 (Mann–Whitney *U* test).

**Figure 4:**
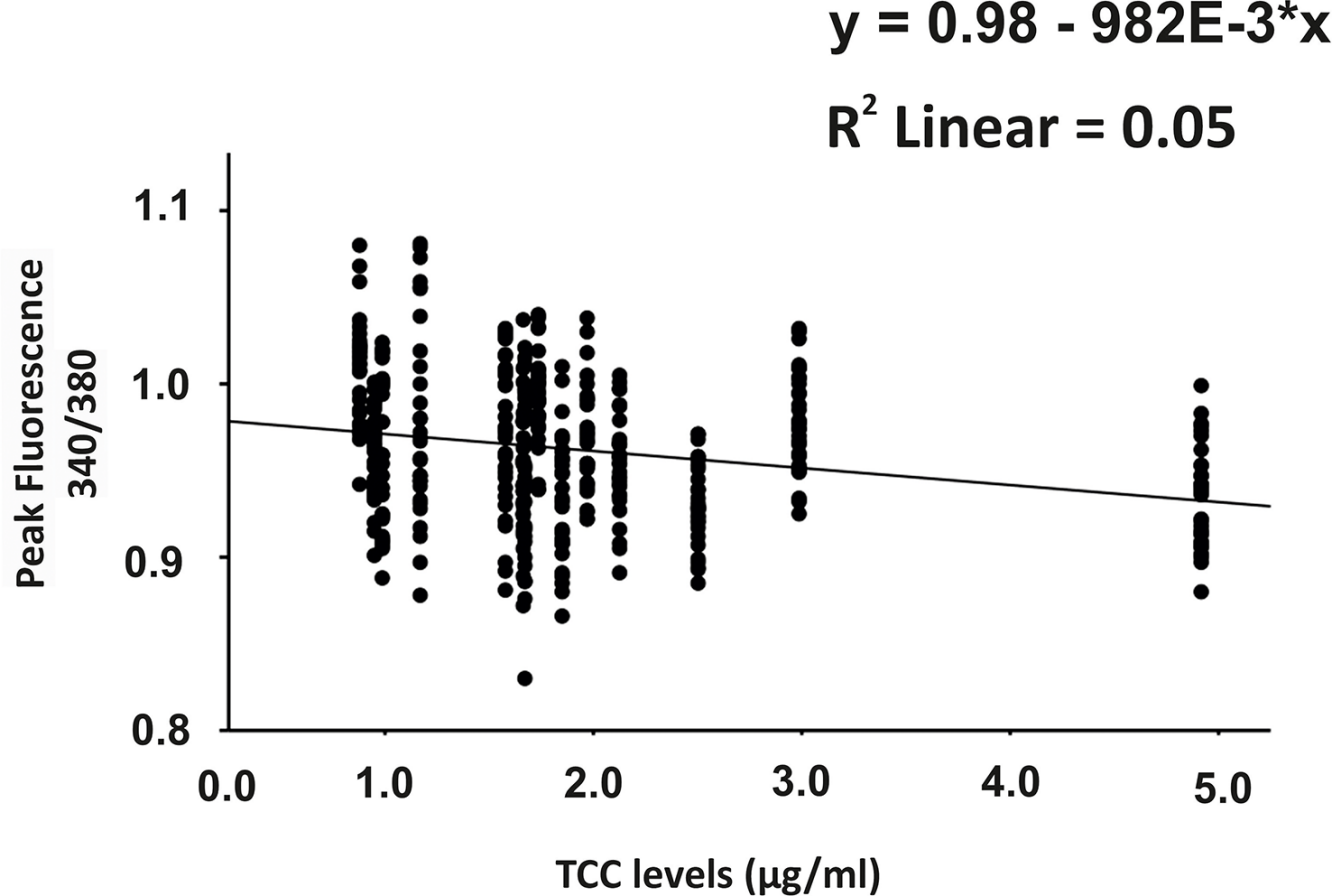
Correlation analysis between plasma terminal complement complex (TCC) levels and the height of the plasma-induced Ca^2+^ peaks. Scatter plot of Ca^2+^ peak induced by patients’ plasma (given in 340/380 fluorescence ratio) over the individual TCC concentrations in the patients’ plasma. Correlation analysis was performed using the generalized estimated equation (GEE) model for longitudinal versus repeated measures (* = multiplies).

Since C6-depleted human plasma indicated a substantial contribution of TCC in a complement-induced Ca^2+^ increase, the plasma from AMD patients versus control plasma was then tested in a similar experiment (**Figure 3**). The waveform of Ca^2+^ transients evoked by AMD patients’ plasma was different from that of control plasma (**Figure 3A**). Although the peak was slightly reduced (**Figure 3B**) and the same level was reached in the sustained phase (**Figure 3C**), the latency was shorter, and the slope of the Ca^2+^ was steeper with a faster time-to-peak (**Figure 3D**) in response to AMD patients’ plasma. To find out whether the patients’ plasma evoked Ca^2+^ increases that resulted exclusively from activated complement proteins, heat-inactivated plasma was used as a control in a similar experiment. Complement as a heat-labile component of the plasma in AMD patients was denatured by incubation at 57°C for 45 min. The heat-inactivated patients’ plasma showed a strongly reduced Ca^2+^ reaction when compared to the untreated plasma from the same AMD patients. The resulting levels did not significantly differ from the baseline before the application of heat-inactivated plasma (**Supplementary Material Figure S2**). These results suggest that the observed differences in the Ca^2+^ signals mainly depended on activated complement components, where some minor effects of other heat-labile plasma compounds cannot be fully excluded. Therefore, a correlation analysis between measured plasma TCC levels and the peak of the single plasma-induced Ca^2+^ signal (**Figure 4**) was performed. For that purpose, TCC levels were compared with the Ca^2+^ amplitude in a scatter plot, and the data were analyzed using a generalized estimating equation (GEE) model. The analysis revealed a significant negative correlation between TCC levels and the Ca^2+^ peak amplitude: the higher the TCC level, the lower the Ca^2+^ peak, which correlates with the significantly lower Ca^2+^ peaks induced by patients’ plasma compared to the controls (**Figures 2**, **3**).

**Figure 5:**
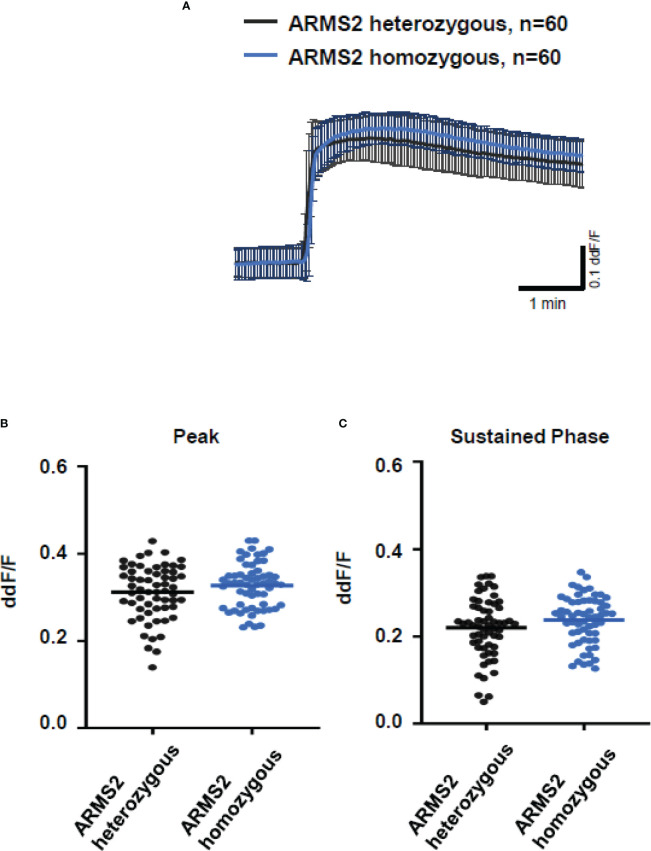
Changes in Ca^2+^ transients activated by plasma from age-related macular degeneration patients, stratified for *age-related maculopathy susceptibility 2 (ARMS2)* risk allele status. Plasma from patients carrying one (heterozygous) *vs.* two (homozygous) *ARMS2* risk alleles (2 different patients per risk allele status, 30 cells per patient). All patients were age-matched and had no additional *Complement factor H* risk alleles. **(A)** Ca^2+^ transients are given as differences to the baseline in fluorescence ratio between the two excitation wavelengths 340 and 380 nm; plasma was used in concentrations of 10%. Data are mean ± SD. **(B, C)** Ca^2+^ transient changes at the initial peak phase and the late sustained phase induced by plasma from patients with one *vs.* two *ARMS2* risk alleles, showing no significant differences in induced Ca^2+^ transients. The horizontal line represents the mean change in Ca^2+^ transients from baseline.

**Figure 6:**
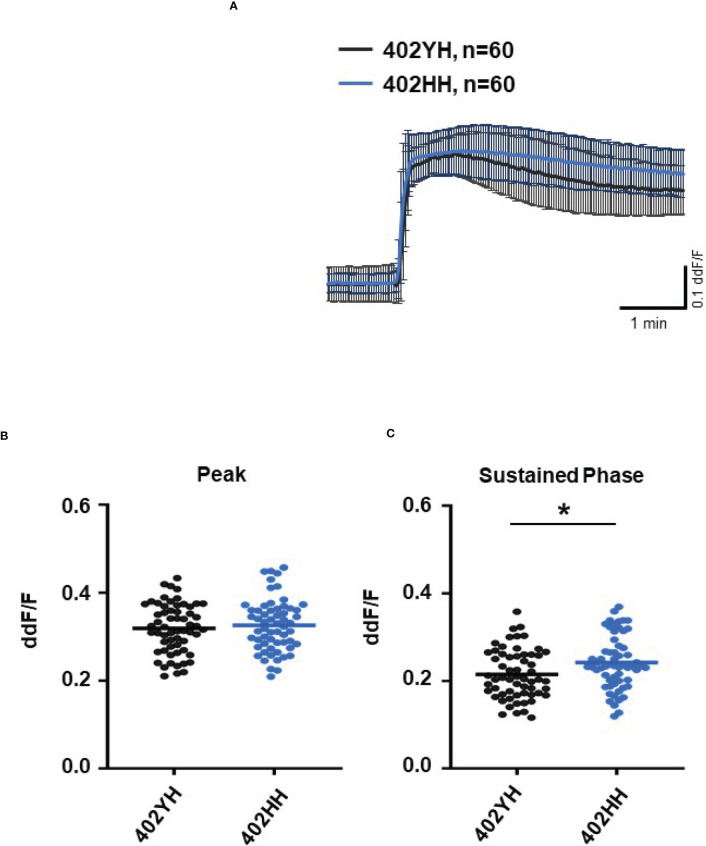
Changes in Ca^2+^ transients activated by plasma from age-related macular degeneration patients, stratified for *Complement factor H (CFH)* risk allele status. Plasma from patients *CFH 402YH* carriers vs. *CFH 402HH* carriers (2 different patients per risk allele status, 30 cells per patient). All patients were age-matched and had no additional *Age-related maculopathy susceptibility 2 (ARMS2)* risk allele. Data are mean ± SD. **(A)** Ca^2+^ transients are given as differences to the baseline in fluorescence ratio between the two excitation wavelengths 340 and 380 nm; plasma was used in concentrations of 10%. **(B, C)** Ca^2+^ transient changes at the initial peak phase and the late sustained phase. Plasma from *CFH 402HH* carriers induced a significantly higher change in intracellular free Ca^2+^ in the sustained phase compared to plasma from carriers of *CFH 402YH*. The horizontal line represents the mean change in Ca^2+^ transients from baseline. *p < 0.05 (Mann–Whitney *U* test).

To further evaluate the role of TCC, we investigated the Ca^2+^ responses from AMD patients’ plasma with defined risk alleles (**Figures 5**, **6**). According to the lack of differences among plasma levels of TCC, we also expected no differences in the Ca^2+^ signals evoked by the patients’ plasma. Indeed, we observed no differences between plasma from heterozygous and homozygous carriers of *ARMS2* risk alleles (**Figures 5A–C**), as well as with plasma from heterozygous or homozygous carriers of *CFH* risk alleles (**Figures 6A–C**). However, we observed subtle differences between the plasma samples of patients with the two investigated risk haplotypes *ARMS2* and *CFH*. We found a higher Ca^2+^ level at the sustained phase of the Ca^2+^ signal in response to plasma of homozygous *CFH 402YY* compared to heterozygous (*CFH 402YH*) individuals, in contrast to plasma derived from patients with the ARMS2 risk allele.

**Figure 7:**
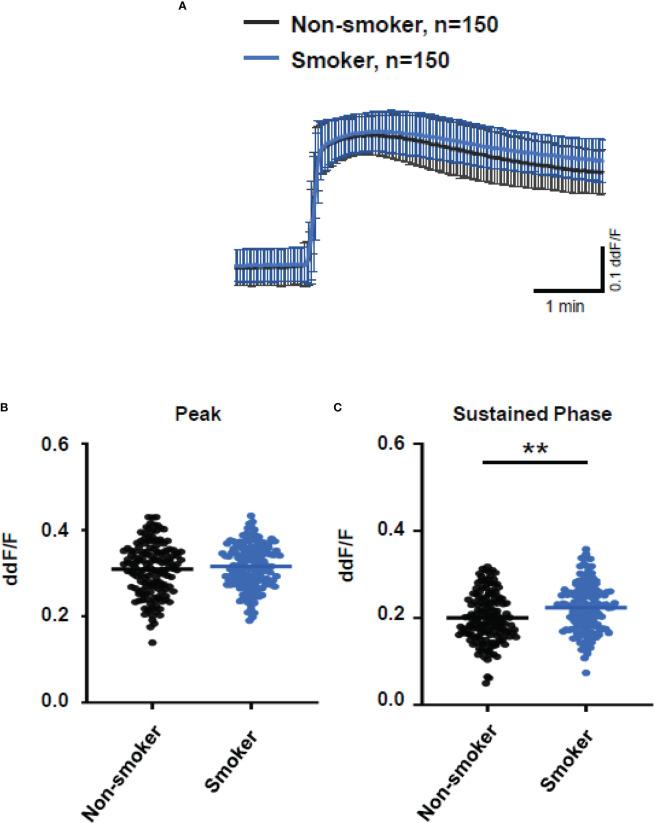
Changes in Ca^2+^ transients activated by plasma from age-related macular degeneration patients, stratified for smoking status (non-smoker *vs.* smoker). Plasma from five different patients per group was compared, with 30 cells per patient. All patients were matched for age, *age-related maculopathy susceptibility 2*, and *complement-factor H* risk alleles. Data are mean ± SD. **(A)** Ca^2+^ transients are given as differences to the baseline in fluorescence ratio between the two excitation wavelengths 340 and 380 nm; plasma was used in concentrations of 10%. **(B, C)** Ca^2+^ transient changes at the initial peak phase and the late sustained phase. Plasma from smoking patients induced a significantly higher change in intracellular free Ca^2+^ in the sustained phase compared to plasma from non-smoking patients. The horizontal line represents the mean change in Ca^2+^ transients from baseline. **p < 0.01 (Mann–Whitney *U* test).

As smoking is a relevant risk factor for AMD, we compared smokers and non-smokers within the AMD patients’ cohort (**Figure 7**). Plasma from smokers and non-smokers induced Ca^2+^ signals in ARPE cells with the same latency and slope of Ca^2+^ increase reaching the same peak. However, plasma from smokers revealed significantly higher sustained phases of the Ca^2+^ signal when compared to that of non-smokers. We observed a comparable effect on the plasma-evoked Ca^2+^ increase with plasma from patients who were homozygous carriers of *CFH 402HH*.

**Figure 8:**
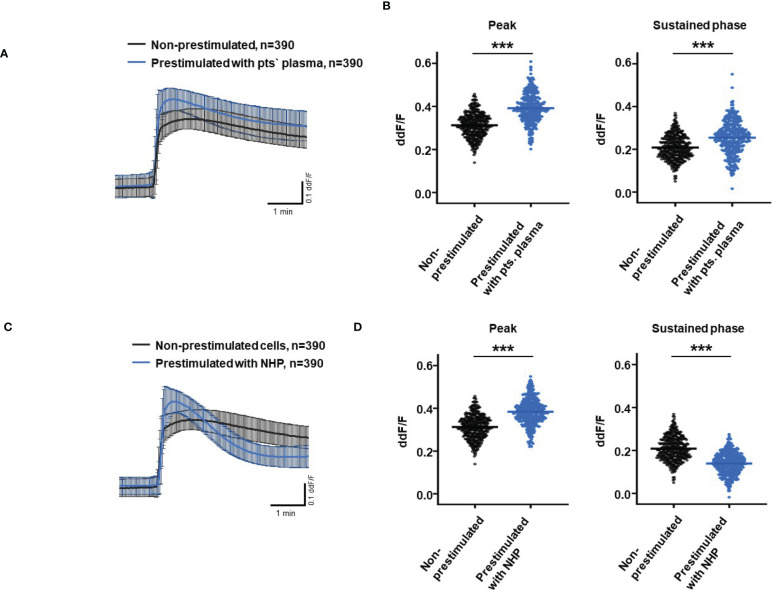
Effects of pre-stimulation with either plasma from age-related macular degeneration patients or controls on Ca^2+^ transients in ARPE-19 cells. The experimental conditions were pre-incubation with serum-free media **(A–D)**, media with 10% patients’ (pts.) plasma (A, B, E, F), or media with 10% normal human plasma (NHP; C–F) for 24 h. Data are mean ± SD. **(A, C)** Ca^2+^ transients are given as differences to the baseline in the fluorescence ratio between the two excitation wavelengths 340 and 380 nm. **(B, D)** Ca^2+^ transient changes at the initial peak phase and the late sustained phase. Cells, pre-stimulated with patients’ plasma, showed a significantly higher change in intracellular free Ca^2+^ compared to non-pre-stimulated cells **(B)**. Cells pre-stimulated with NHP showed a significantly higher change in intracellular free Ca^2+^ at the initial peak phase but a significantly lower amplitude in the sustained late phase compared to non-pre-stimulated cells **(D)**. The horizontal line represents the mean change in Ca^2+^ transients from baseline. ***p < 0.001 (Mann–Whitney *U* test). The dataset for “non-prestimulated” for “peak” and “sustained” were statistically tested two times: Once against “pre-stimulation patients’ plasma” and a second time against “pre-stimulated NHP”; thus, the data for “non-stimulated” “peak” and “sustained” in the **(B, D)** are identical.

**Figure 9:**
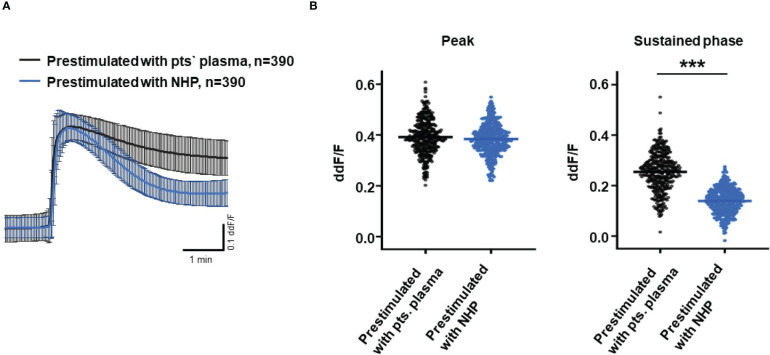
Direct comparison of pre-stimulation effects between patients’ plasma and control plasma on Ca^2+^ transients in ARPE-19 cells. **(A)** Ca^2+^ transients are given as differences to the baseline in the fluorescence ratio between the two excitation wavelengths 340 and 380 nm; plasma was used in concentrations of 10%. Data are mean ± SD. **(B)** Compared to pre-stimulation with normal human plasma (NHP), cells showed a significantly higher change in intracellular free Ca^2+^ in the sustained phase after pre-stimulation with patients’ plasma. The horizontal line represents the mean change in Ca^2+^ transients from baseline. ***p < 0.001 (Mann–Whitney *U* test).

**Figure 10:**
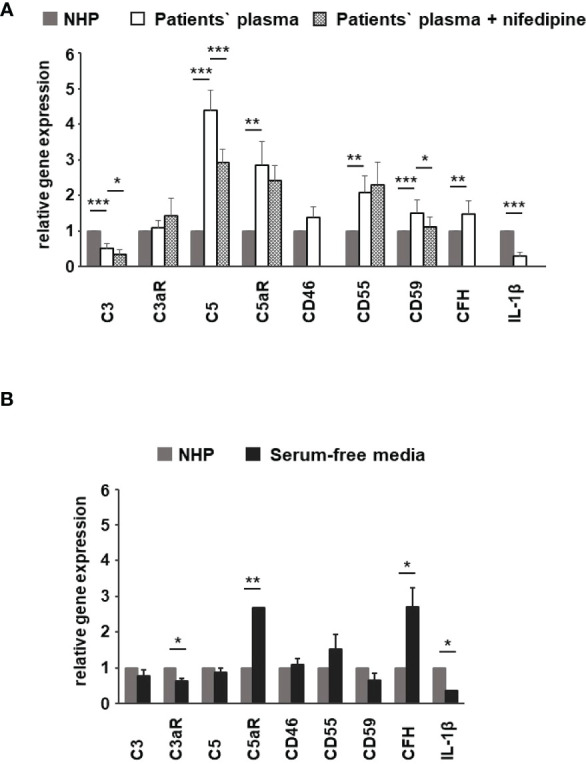
Effects of patients’ plasma on differential gene expression of complement genes in ARPE-19 cells. Effect of patients’ plasma ± nifedipine **(A)** and serum-free media **(B)** and plasma compared to normal human plasma (NHP; 10%) on gene expression of C3, C3aR, C5, C5aR, CD46, CD55, CD59, complement factor H (*CFH*), and interleukin-1beta (IL-1β) in ARPE-19 cells. NHP and patients’ plasma were applied at a total concentration of 10% with nifedipine at 10 µM for 24 h. Data are expressed as mean values + SD. For serum-free media, n = 2–3. For patients’ plasma, n = 16, except for C5aR, n = 6. Patients’ plasma + nifedipine, n = 9, except for C5aR, n = 5. For NHP, n = 3. *p < 0.05, **p < 0.01, ***p < 0.001 (Student’s t-test).

So far, our experimental results suggest a potential impact of TCC on the RPE. To mimic the effects of chronic complement stimulation, we pre-incubated ARPE-19 monolayers for 24 h with patients’ plasma before we measured Ca^2+^ increases (**Figure 8**). Pre-stimulation with patients’ plasma led to a marked increase in evoked Ca^2+^ transients (**Figure 8A**) with significantly higher peaks and sustained phases (**Figure 8B**). As the patients’ plasma contents caused ARPE-19 cells to be more sensitive to complement, this “pathological” effect was further analyzed by varying the pre-incubation conditions and using control plasma (NHP) for pre-incubation of the cells. In addition, pre-incubation with NHP changed the Ca^2+^ transients evoked by patients’ plasma but in a different way (**Figure 8C**). Again, the peak was significantly increased, but the sustained phase was markedly reduced (**Figure 8D**). Normal human plasma seemed to lack those components, which were required for complement sensitization. This was also seen in the comparison of the subsequently evoked Ca^2+^ transients with patients’ plasma after pre-incubating the cells with either standard control plasma or patients’ plasma (**Figure 9A**). The peaks from both pre-incubation conditions looked very similar, but the sustained phase of cells pre-incubated with patients’ plasma was substantially higher than that of cells pre-incubated with normal humans (**Figure 9B**).A central paradigm in Ca^2+^ signaling is that Ca^2+^ signals specify the cell function changes by its waveform and spatial distribution as a code (50–52). Thus, differences in the waveforms have different effects on the cell’s function. These waveform differences might change intracellular signaling and gene expression profiles. Therefore, gene expression activities in ARPE-19 cells stimulated by patients’ plasma and control plasma were quantified, as well as cells that were kept overnight under plasma-free conditions (**Figure 10**), thereby concentrating our analysis on genes of the complement system, the pro-inflammatory cytokine IL-1β, and the surface receptors CD46 (cleavage of C3b and C4b), CD55 (accelerated decay of complement proteins), and CD59 (TCC formation inhibition).

To identify the contribution by L-type channels, nifedipine (10 µM), an L-type Ca^2+^ channel blocker, was used. Incubation of cells with patients’ plasma resulted in an upregulation of C5, C5aR, CD55, CD59, and CFH (**Figure 10A**). In parallel, C3 and IL1-β were downregulated (**Figure 10A**). The differential regulation by patients’ plasma of C3, C5, C5aR, and CD59 was nifedipine sensitive, indicating that these expression changes depend on complement action, with a great likelihood of the presence of TCC. Comparing standard control plasma with plasma/plasma-free conditions, we found that the ARPE-19 cell reacted with a selective downregulation in the C5aR 1 (there are two C5a receptors) and CFH expression but an increase in the expression of C3aR and IL-1β (**Figure 10B**) in response to control plasma.

**Figure 11:**
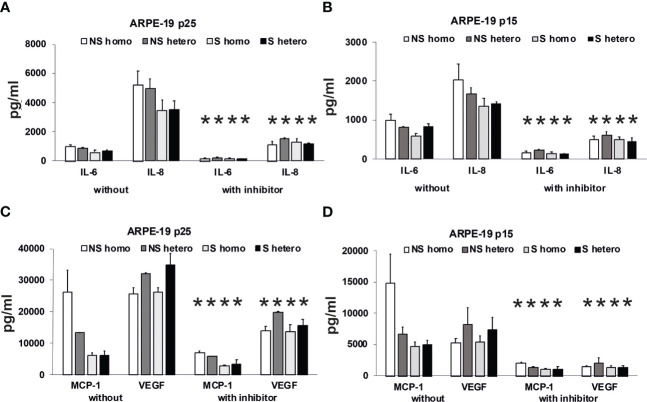
Effects of plasma on differential secretion activities of ARPE-19 cells. Cytokine and chemokine secretion of ARPE-19 cell lines. **(A, C)** ARPE-19 in passage 25. **(B, D)** ARPE-19 in passage 15. **(A–D)** Plasma (all 10%) from non-smoking (NS) or smoking (S) age-related macular degeneration (AMD) patients, carriers *CFH 402HH* versus carriers *CFH 402YH*. Plasma samples with or without PI3K-inhibitor LY294002 (50 µM) were incubated with ARPE-19 cells and cyto-/chemokine concentrations determined from culture supernatants. n = 2–4 for CHF mutant plasma (* = p< 0.05 for all respective values of supernatants from cultures with inhibitor vs. without).

To substantiate the hypothesis that the patients’ plasma promotes an immune stimulatory phenotype of RPE cells, we analyzed the secretory profile of ARPE-19 cells under 3 days of stimulation with AMD patients’ plasma (**Figure 11**). Here, we used plasma from patients heterozygous (*CFH 402YH*) or homozygous (*CFH 402HH*) for the *CFH* risk allele who were additionally differentiated by their smoking status. In a third, independent assay, we tested plasma pools from elderly patients of different age groups but without AMD for the induction of cytokine secretion by ARPE-19 cells (see **Supplementary Material**).

Using the multiplex technology, we analyzed a broad profile of immune and angiogenesis-relevant factors. We plotted only those factors that were induced by patients’ plasma in ARPE-19 cell lines from two different laboratories: one in passage 25 (**Figures 11A, C**) and the other in passage 15 (**Figures 11B, D**). In general, we found induction of IL-6, IL-8/CXCL8, MCP-1/CCL2, and VEGF-A, with the predominant secretion of VEGF-A and MCP-1, while the other tested cytokines, IL-1β, IL-1RA, IL-10, IL-12(p70), and IFN-γ, were not detected.

Among the risk types with homozygous *CFH* deficiency (*CFH 402HH*) or smoker status, we found comparably strong induction of cytokine secretion. However, an exception appeared to be the group of non-smokers with *CFH* polymorphisms. In this group, we found increased MCP-1 levels compared to the smoker group, especially when the donors were homozygous for *CFH 402HH*. The MCP-1 secretion was twice as high as in the heterozygous *CFH 402YH* non-smoker group and increased three- to fourfold compared to the group of smokers with *CFH 402HH* risk polymorphism. Concerning VEGF secretion, homozygous *CFH 402HH* donors, irrespective of their smoking status, displayed a slightly decreased secretion when compared to the heterozygous groups. The addition of the PI3K inhibitor resulted in a significantly reduced secretion to less than 50%, while the pattern of secretion remained the same.

In our experiments, ARPE-19 cells of the higher passages 15 and 25 secreted higher levels of cytokines than those of passage 6 (**Supplementary Material Figure S3**), potentially reflecting the situation of aged RPE.

## 4 Discussion

As polymorphisms in complement genes are associated with the risk of AMD, it is questioned whether this leads to systemic or only local effects. Chirco et al. (17) did not find a difference in TCC levels in the plasma of AMD patients with low-risk and high-risk *CFH* alleles, whereas in the retinas of patients with high-risk *CFH* alleles, higher local TCC concentrations were detected. The current literature indicates an important role of TCC in the etiology of AMD in two ways. On the one hand, systemic changes by increased complement activity and higher TCC levels in AMD patients are discussed, as well as higher risks for AMD associated with polymorphisms in C9. On the other hand, the exploration of complement affecting RPE cells at a cellular level followed the concept of “sub-lytic” MAC influencing cell function. Our study provides a direct link between the mechanistic levels: the patients’ observations and the cellular effects of isolated TCC on RPE cells. The most relevant observation of our study is that AMD patients showed higher plasma levels of TCC when compared to age-matched controls, but there were no differences among carriers with different risk alleles of complement genes. These systemic changes affect the Ca^2+^ signaling that regulates the expression activity of complement genes in the RPE and thus the control of the local activity of the complement system.

In our study, we investigated the TCC levels from the plasma of AMD patients and compared them with those from the plasma of AMD patients and age-matched controls as well as carriers of risk alleles *CFH* and *ARMS2*. Whereas AMD patients’ plasma showed higher TCC levels, there were no differences between plasma from AMD patients with either *CFH* or *ARMS2* risk alleles. The same applies to the comparison between smokers and non-smokers in the patients’ cohort. This observation matches well with that from other publications. First, our data confirmed the conclusions by Chirco et al. (17), who found no differences in TCC levels in the plasma between carriers of polymorphism *CFH 402HH* and *CFH 402YY* controls. Thus, the increased TCC levels in the plasma correlate with the diagnosis of AMD and, thus, clinically relevant degenerative changes in the retina. This assumption would also explain why there are no differences between AMD patients in the comparison of genetic and smoking-associated risk profiles. In the measurement of complement activity markers in the plasma, such as C3d/C3 ratio, C3a-desarg, or TCC, the different risk allele *CFH*, *ARMS*, or *CFI* carriers display comparable levels of those markers, while healthy donor’s plasma displayed increased levels of complement activity (18, 19, 21, 23, 53–58). Increased markers for complement activity were found when compared to those in control plasma in both patients with dry and wet AMD. Our study included patients with wet AMD showing that not only dry AMD patients are affected by increased complement activity in the plasma. In contrast to these data, the C9 risk alleles have differential effects on TCC or C9 plasma levels (19, 20, 58). C9 risk allele carriers show higher levels of TCC when comparing AMD versus non-AMD patients, and also among AMD patients, the carriers of the C9 risk allele have higher TCC levels than non-carriers, which also varies within the group of C9 risk allele carriers depending on specific polymorphisms. Thus, considering our data and data from existing literature, we conclude that increased TCC or C9 levels in the plasma are associated with the disease AMD. However, the increased plasma levels originate at different steps of the complement cascade: either at the insufficient control of the alternative activation pathway or directly by determining the gain-of-function effects by altered C9 proteins. In summary, our data further support the prominent role of the TCC in the etiology of AMD.

To determine whether these differences in plasma TCC levels are of biological relevance, we incubated these plasma probes with ARPE-19 cells. Although this RPE cell line is under debate for being representative of the native RPE and might thus limit our conclusions for the pathogenic mechanisms possibly taking place *in vivo*, these cells will reflect the differences in the biological activity of patients’ plasma. As in many other and also in our own recent studies, we used the Ca^2+^ imaging technique to monitor the cell reactions to plasma as a complement resource (28, 35, 59). We know from our own studies that activated complement compounds produced by the complement cascade induced by human plasma evoke orchestrated Ca^2+^ signals by activation of endogenously expressed ion channels (28). The central paradigm for coding the desired specific change of cell function activated by an increase in intracellular free Ca^2+^ states its origins in the time-dependent shape and the spatial distribution of the signal (50–52). Thus, differences in shape and distribution represent different changes in cellular functions.

Considering the importance of the shape of Ca^2+^ transients to represent specific cellular functions (50–52) and taking into account the reproducibility of complement-evoked Ca^2+^ increases (28, 33), we tested the effects of patients’ plasma on intracellular free Ca^2+^ as a second messenger. Before we used patients’ plasma, we tested C6-depleted plasma to identify the parts of the Ca^2+^ signal under the influence of TCC. The Ca^2+^ signal evoked by C6-depleted plasma was strongly reduced in peak amplitude and showed an absence of a sustained phase. Thus, TCC that is formed in human plasma during the first seconds of exposure to the cells already determines the first phase of the Ca^2+^ increase and additionally paves the full development signal. As the later parts of the plasma-induced Ca^2+^ are activated by anaphylatoxins (28, 33), the early increase by TCC is of importance for anaphylatoxin signaling. In a recent study, we showed that isolated anaphylatoxins exhibit monophasic Ca^2+^ transients with amplitudes (33) that are far smaller than those of complete plasma, which shows biphasic Ca^2+^ transients consisting of an initial peak and a sustained phase (28). Thus, the C6-depleted plasma indicates that especially the early increase of intracellular Ca^2+^ stems from the presence of TCC.

Indeed, comparing Ca^2+^ transients evoked from control plasma to those evoked from patients’ plasma, we found that the Ca^2+^ transients from AMD plasma increased faster with shorter latency and a steeper increase to a slightly reduced peak, whereas the sustained phases remained unchanged. For the peak level of the Ca^2+^ signals, we found a negative correlation with the TCC levels in the plasma of individual patients. Thus, the plasma with higher levels of TCC especially showed changes in the first phase of the signal as hypothesized from the data with C6-depleted plasma. Heat inactivation of patients’ plasma to inhibit complement activity revealed no increased intracellular free Ca^2+^, which suggests that the Ca^2+^ signal from patients’ plasma depends on the activity of complement, although the minor contribution of other heat-labile factors cannot be fully excluded. Indeed, we found a correlation between the plasma TCC levels in the patients and the peak amplitude of the Ca^2+^ increase, evidencing that the major differences in the Ca^2+^ signals between control plasma and patients’ plasma result from the differences in the plasma TCC levels. Thus, any changes resulting in Ca^2+^ signals and also changes in gene expression should be attributed to the higher TCC levels in the patients’ plasma.

In general, we found no differences in the plasma TCC levels between the carriers of risk alleles *ARMS2* and *CFH*. However, when comparing the plasma-evoked Ca^2+^ signals from carriers of different risk alleles, we observed subtle differences. With *ARMS2* genotypes, the Ca^2+^ signals were indistinguishable between heterozygous and homozygous carriers, while the *CFH 402HH* carrier’s plasma showed higher amplitudes in the late phase than plasma from *CFH 402YH* individuals. We observed the same when comparing plasma from smokers with non-smokers. In addition, only the late phase was increased. A decreased CFH efficiency to control the alternative complement pathway by either polymorphic CFH or by cigarette smoke (3, 6, 7, 44, 60–62) leads to increased complement activity markers in the patients’ sera (57, 60, 63). Because of this, we assume that sera of smokers or patients carrying *CFH* risk alleles contain higher levels of anaphylatoxins, which in turn increase the late Ca^2+^ signals. The study by Smailhodzic et al. supports this conclusion (21). The study reports that sera from *CFH* risk allele carriers show higher systemic complement activity determined as C3d/C3 ratio compared to *ARMS2* risk allele carriers. However, we found no differences in the TCC levels in the plasma of mutated *CFH* and *ARMS2* allele carriers. We explain this difference by the fact that C3d/C3 measurements reflect a more dynamic parameter based on complement factors that are more unstable than TCC.

Given the above conclusions, the Ca^2+^ transients evoked in ARPE-19 cells by plasma as a complement source represent integrals of the biological activity of activated complement. To shift these observations toward more translational conclusions, we performed pre-stimulation experiments with plasma to mimic a sustained exposure to complement as it likely occurs in the patients’ eye. After pre-incubation with patients’ plasma, both the peak and sustained phases are higher than those without pre-stimulation. Thus, the pro-inflammatory complement composition in the patients’ sera sensitizes ARPE-19 cells for complement reactions. In contrast, after pre-stimulation with control plasma, the patients’ sera show a differentially regulated response. Although the peak increases in the same manner, the sustained phase is much smaller in amplitude. Thus, control plasma also sensitizes the cells for the initial reaction but leads to a faster termination of the Ca^2+^ signal. The composition of activated complement in control sera maintains the immune inhibitory activity of RPE cells against the pro-inflammatory complement activity of patients’ sera.

To support this conclusion, we investigated gene expression profiles of ARPE-19 cells in response to stimulation by control and patients’ sera. The RPE cell reaction to complement under healthy conditions includes the secretion of CFH to prevent local complement reactions at the moment they would occur (44, 62–66). Thus, we investigated the effects of the patients’ sera on complement gene expression in ARPE-19 cells. In previous publications, we have shown that the complete Ca^2+^ signal evoked by complement is blocked by the inhibition of L-type channels (28). Furthermore, the steepness of the Ca^2+^ increase was profoundly reduced. This matches well with the kinetic alterations associated with patients’ plasma that contains higher levels of TCC. However, when investigating the Ca^2+^ signals evoked by isolated anaphylatoxins, we found that these Ca^2+^ signals were insensitive to L-type channel blockers (33). Thus, the blocking effects of the L-type channel blocker nifedipine indicate the contribution of TCC to gene expression. We compared gene expression using control plasma versus plasma-free conditions and observed a decrease in the C3aR expression, in parallel to an increase in C5aR and CFH expression. Furthermore, the IL-1β expression decreased. This effect on IL-1β expression might result from anaphylatoxin C5a as shown by Brandstetter et al. (67), who also reported that the expression profile induced by C5a increased further inflammasome priming by IL-1β. When studying the effects of the isolated anaphylatoxins, we found no effects on the C3, C5, or anaphylatoxin receptor expression (33). Thus, the additional presence of TCC is required to produce the differential gene expression changes as shown by Brandstetter et al. (67), and in our study, such an interactive effect between activated complement components that we have previously described (33) demonstrates interactive signaling of C3a and C5a. In combination with our new data, the picture is widening to an effect in which control plasma causes the cell reactions to have a higher sensibility for the C3 convertase level: more signaling with C3aR and increased C3 convertase activity by CFH downregulation and likely less C5aR1 and C5aR2 signaling. In contrast, with patients’ plasma incubation, the expression of C3 decreased, but C5 is now more strongly expressed. This goes along with increases in C5aR1 expression and increased expression of TCC surface inhibitors CD55 and CD59. Thus, in contrast to control plasma, AMD plasma shifts the cell activity toward the C5 convertase level with higher C5a signaling and preparation for higher levels of TCC. Compared to control plasma, IL-1β expression is further decreased. These observations and the observations of Brandstetter et al. (67) led us to the conclusion that the patients’ plasma turns the expression profile into a more pro-inflammatory phenotype in ARPE-19 cells. The reduction in C3 expression along with increases in C5 and CD59 expression is sensitive to the L-type channel blocker nifedipine and thus induced under contribution by TCC. With an increased production of C5, the RPE would at the same time lead to more formation of TCC and thus increase its protection against TCC impact. Our observations of the secretory activity under the influence of patients’ plasma support this conclusion. Also, under treatment with AMD plasma, ARPE-19 cells showed increased secretion of pro-inflammatory cytokines IL-1, IL-6, IL-8, MCP-1, and the angiogenic factor VEGF-A; among them, the secretion of MCP-1 and VEGF-A was the highest. Interestingly, we found differences between smokers and non-smokers for MCP-1 secretion. The non-smoker group showed generally higher MCP-1 secretion rates with differences between the *CFH* risk haplotypes when compared to the smoker group, in which we also found no differences between the *CFH* haplotypes. This corresponds to the differences in the Ca^2+^ signal patterns. The comparison between smokers and non-smokers showed no differences in the peak, only in the sustained phase, whereas the peak was dependent on the TCC concentration. This indicates that there is no general pattern of secretion activity associated with the different risk factors, but, in general, the risk factors lead to increased secretion of pro-inflammatory cytokines. Thus, under the influence of TCC, the RPE’s phenotype is changing into a pro-inflammatory one including a self-protection of the RPE against TCC.

Here, we have investigated the reaction of ARPE-19 cells to AMD patients’ plasma, which indicated a biological impact on the cells, rendering the immune reactions toward a more pro-inflammatory type. Although the ARPE-19 cell line might not reliably represent properties of RPE cells *in vivo*, we can principally draw conclusions on the capabilities of the patients’ plasma themselves. Here, the main conclusion is that in AMD, a systemic impact like the complement system acts on vulnerable cells of the outer blood–retina barrier. AMD risk alleles exacerbate local immune reactions, and the systemic pre-activated complement system might affect the outer retina even without local complement activity. The TCC might play an important role among the systemic factors, leading to a local effect on the tissue, which might not be primarily fatal for RPE cells. We assume that this effect drives the chronic low-grade inflammation known to occur in AMD patients and is reflected by observations such as a life-long accumulation of TCC in the outer retina (15–17) by increased levels of terminal complement complex in the blood (20, 21, 68).

## Data availability statement

The original contributions presented in the study are publicly available. This data can be found here: https://doi.org/10.5281/zenodo.7797381 or DOI 10.5281/zenodo.7797381 (Zenodo repository).

## Ethics statement

The studies involving human participants were reviewed and approved by Charité ethics committee, registration number EA2/004/14. The patients/participants provided their written informed consent to participate in this study.

## Author contributions

Conceptualization: CB, PZ, AJ, CS and OS. Methodology: CB, SR, AS, LP, CH and MG. Formal analysis: CB, SR, LP, CS, GW and OS. Investigation: CB, SR, AS, LP, CH and MG. Writing—original draft preparation: OS, CB, CS and GW. Writing—review and editing: OS, SC, GW and JP. All authors contributed to the article and approved the submitted version.

## Supplementary material

Supplementary Figure 1 Tables of patients’ and risk factors. Upper table shows the age and gender distribution in the patients cohort and the group of healthy donors. The lower table shows the distribution of risk factors among the AMD patient cohort.

Supplementary Figure 2 Properties of patients whose plasma was used for Ca^2+^ imaging, qPCR and secretion assay. Table shows detailed properties of the patient donors whose plasma was used for biological analyses. Note: the numbers are not suitable to identify certain patients.

Supplementary Figure 3 Heat-inactivation control for patient’s plasma. Changes in Ca^2+^ transients activated by plasma from age-related macular degeneration patients (16 different patients, 30 cells per patients) and heat-inactivated patients’ plasma (16 different patients, 10 cells per patients). Ca^2+^ transients are given as differences to the baseline in fluorescence ratio between the two excitation wavelengths 340 nm and 380 nm. Data are mean ± SD.

Supplementary Figure 4 Complement-dependent activation of basal secretion by ARPE-19 cells. ARPE-19 cells in passage 6 were stimulated with normal human plasma (NHP, 10%) from age-matched, non-smoking donors without AMD, *CFH* mutations unknown. Secretion of different cytokines was measured in supernatants with a multiplex bead assay; cytokine concentrations are given as mean + SEM; n = 17. In a second, independent experiment, pooled plasma samples of age-matched donors without AMD were tested for their ability to induce cytokine secretion by ARPE-19 cells in their sixth passage. In this experiment, the plasma pools displayed slight differences in their ability to induce cytokine secretion in ARPE-19 cells, the “oldest” pool (mean donor age: 82.7 years) induced more IL-6 and IL-8 and less MCP-1 compared to the pools of younger donors (means of 77.4 and 73 years, respectively). Data not shown. Interestingly, the used plasma pools already contained a high concentration of MCP-1, which decreased fivefold during the incubation with ARPE-19 cells, suggesting that MCP-1 was obviously used by the ARPE-19 cells, while IL-8 was produced by the RPE cells themselves and not provided by the plasma. VEGF concentrations remained unaltered, not allowing any conclusion on use or production by the ARPE-19 cells.
