# Supplementary figures and images for: Increased plasma level of terminal complement complex in AMD patients: potential functional consequences for RPE cells
Source: Front Immunol. 2023 Jun 8;14:1200725. doi: 10.3389/fimmu.2023.1200725 (PMC10287163; doi:10.3389/fimmu.2023.1200725)

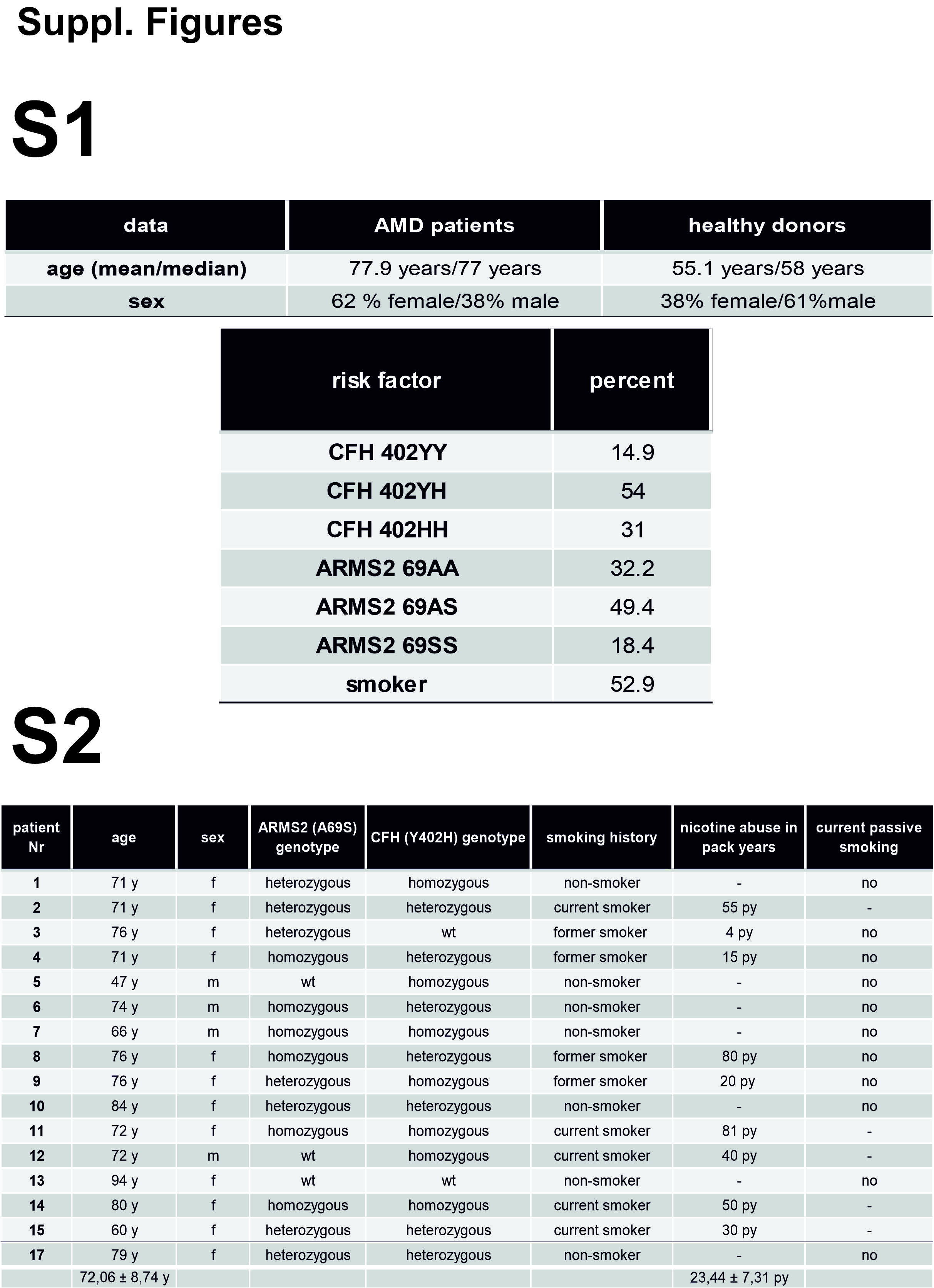

Supplement: Supplementary Figure 1 — Tables of patients’ and risk factors. Upper table shows the age and gender distribution in the patients cohort and the group of healthy donors. The lower table shows the distribution of risk factors among the AMD patient cohort. [file Image_1.jpeg]

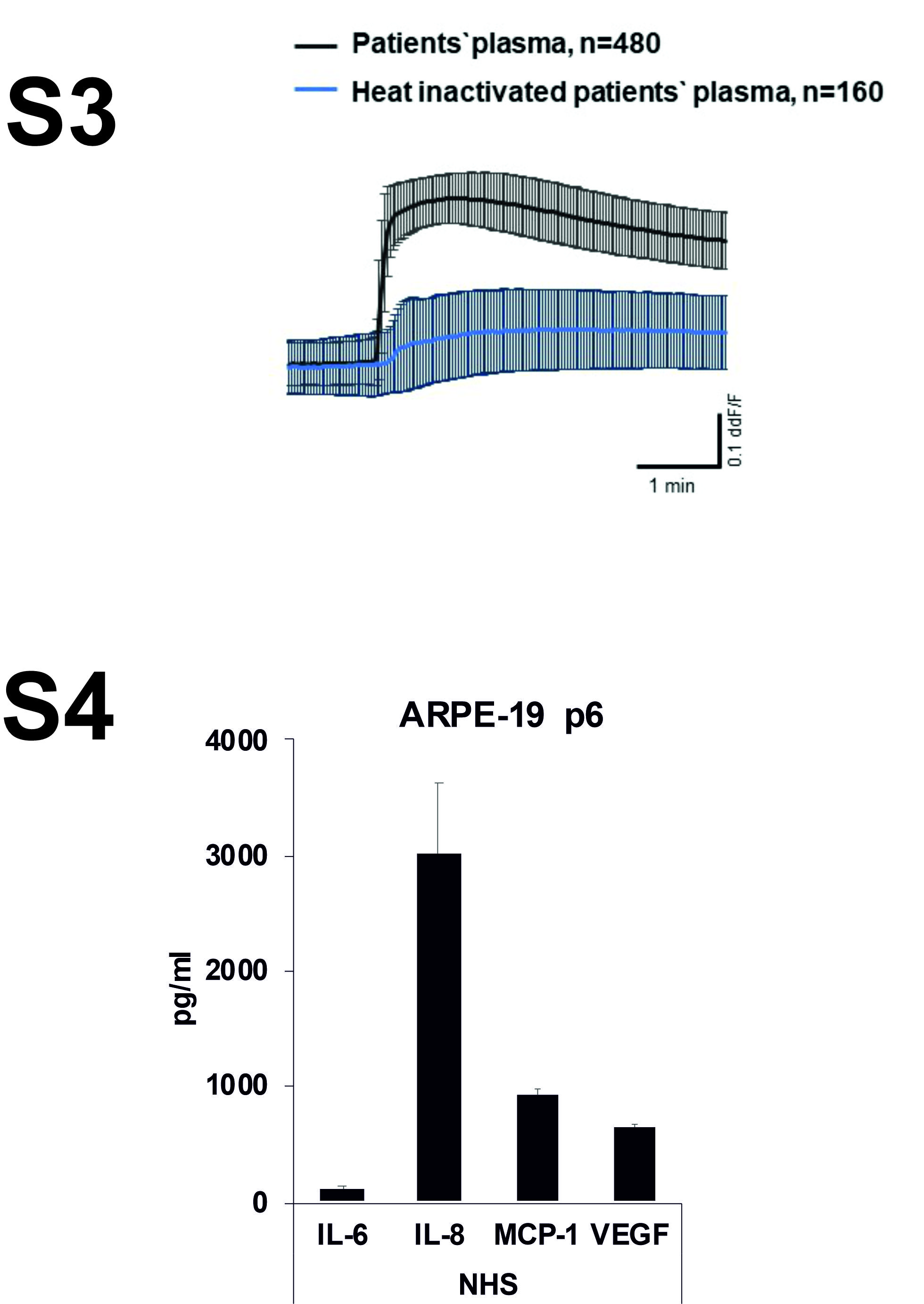

Supplement: Supplementary Figure 3 — Heat-inactivation control for patient’s plasma. Changes in Ca2+ transients activated by plasma from age-related macular degeneration patients (16 different patients, 30 cells per patients) and heat-inactivated patients’ plasma (16 different patients, 10 cells per patients). Ca2+ transients are given as differences to the baseline in fluorescence ratio between the two excitation wavelengths 340 nm and 380 nm. Data are mean ± SD. [file Image_2.jpeg]
